# Cascading detection model for prediction of apnea-hypopnea events based on nasal flow and arterial blood oxygen saturation

**DOI:** 10.1007/s11325-019-01886-4

**Published:** 2019-07-05

**Authors:** Hui Yu, Chenyang Deng, Jinglai Sun, Yanjin Chen, Yuzhen Cao

**Affiliations:** 1 Department of Biomedical Engineering, Tianjin University, Tianjin, China; 2 Tianjin Hospital of ITCWM Nankai Hospital, Tianjin, China

**Keywords:** Sleep apnea and hypopnea syndrome, Apnea-hypopnea index, Polysomnography, Cascading detection model, Apnea-hypopnea events

## Abstract

**Purpose:**

Sleep apnea and hypopnea syndrome (SAHS) seriously affects sleep quality. In recent years, much research has focused on the detection of SAHS using various physiological signals and algorithms. The purpose of this study is to find an efficient model for detection of apnea-hypopnea events based on nasal flow and SpO_2_ signals.

**Methods:**

A 60-s detector and a 10-s detector were cascaded for precise detection of apnea-hypopnea (AH) events. Random forests were adopted for classification of data segments based on morphological features extracted from nasal flow and arterial blood oxygen saturation (SpO_2_). Then the segments’ classification results were fed into an event detector to locate the start and end time of every AH event and predict the AH index (AHI).

**Results:**

A retrospective study of 24 subjects’ polysomnography recordings was conducted. According to segment analysis, the cascading detection model reached an accuracy of 88.3%. While Pearson’s correlation coefficient between estimated AHI and reference AHI was 0.99, in the diagnosis of SAHS severity, the proposed method exhibited a performance with Cohen’s kappa coefficient of 0.76.

**Conclusions:**

The cascading detection model is able to detect AH events and provide an estimate of AHI. The results indicate that it has the potential to be a useful tool for SAHS diagnosis.

## Introduction

Sleep apnea and hypopnea syndrome (SAHS) is a prevalent sleep breathing disorder in middle-aged people. The gold standard for diagnosis of SAHS is to perform polysomnography (PSG) in a laboratory. However, PSG requires patients to sleep with many sensors for at least one night; the scoring of apnea-hypopnea (AH) events can take a long time. Therefore, many researchers hope to simplify or replace PSG by using a limited number of physiological signals. Electrocardiogram (ECG) was first studied for this purpose. McNames et al. [1] found that heart rate, S-pulse amplitude, and pulse energy were correlated with SAHS. Bsoul et al. [2] cut the ECG into 60-s segments and used a support vector machine (SVM) for real-time detection of apnea. However, many other diseases except SAHS also affect ECG. Hence, nasal flow (NF) [3–6], arterial blood oxygen saturation (SpO_2_) [7], snoring [8], or a combination of these signals [9, 10] have been adopted more recently. Gutierrez et al. [4] used the overall features of NF for the diagnosis of SAHS severity. Xie et al. [10] utilized a combination of classifiers to achieve real-time detection of SAHS based on ECG and SpO_2_. All the above studies can be roughly divided into two categories: those that predict the AH index (AHI) based on the detection of AH events [2, 3, 5, 7, 9–11], and those that predict AHI based on the overall signal features [1, 4, 6, 8, 12, 13]. The latter approach cannot provide time information for each AH event, whereas most studies in the former [2, 7, 10, 11] only involve a 60-s segment identification which may not be accurate for predicting the segments containing multiple AH events and may lead to errors in the estimation of AHI. On the other hand, the methods mentioned above include rule-based [5, 7, 9], SVM [2, 10, 11], and supervised neural network [3, 11], which require a large number of hyperparameters to be set by experience. Therefore, we utilized random forests composed of classification and regression trees (CARTs) based on morphological features extracted from NF and SpO_2_ for AH events detection. A 60-s detector and a 10-s detector were cascaded for more precise detection of AH events.

## Materials and methods

### Subjects

The St. Vincent University Hospital/Dublin University College Sleep Apnea Syndrome Database (UCDDB) [14] public on Physionet [15] was used for a retrospective data analysis throughout this paper. The database contains 25 subjects’ PSG data, including EEG, electrooculogram, submental electromyography, NF, ribcage and abdomen movements, SpO_2_, snoring, and body position. All signals were obtained using a Jaeger–Toennies system. The annotation files consisted of onset time and duration of respiratory events provided by an experienced specialist. The cutoff values for AHI were commonly set to 5, 15, or 30 events/h [3, 4, 7, 16, 17]. There were data for two non-SAHS subjects, twelve mild-SAHS subjects, five moderate-SAHS subjects, and six severe-SAHS subjects in the database. While there was a severe distortion in the NF signal of subject ucddb005 thus this recording was excluded. Consequently, totally 24 subjects’ polysomnography recordings were taken into this study. The sleep-related parameters of the subjects are summarized in Table 1.

**Table 1:** Summary of sleep-related parameters (mean ± standard deviation)

	Non-SAHS	Mild SAHS	Moderate SAHS	Severe SAHS
Number of patients	2	12	5	5
Age (years)	52.0 ± 15.6	48.6 ± 8.5	56.8 ± 6.4	46.6 ± 5.5
AHI (events/h)	4.1 ± 5.7	9.9 ± 2.9	24.6 ± 3.9	43.8 ± 16.3
Epworth Sleepiness Score	7.0 ± 8.5	11.6 ± 5.1	11.2 ± 6.9	12.4 ± 7.9

According to the American Academy of Sleep Medicine (AASM) manual [16], apnea is scored when there is a more than 90% drop in the peak signal of the pre-event baseline for NF with a duration longer than 10 s. Hypopnea is scored by the following rules: (1) there is a more than 30% drop in the peak signal of the pre-event baseline for nasal pressure with a duration longer than 10 s, accompanied by (2) more than 3% arterial oxygen desaturation or an arousal. As a result, we selected NF and SpO_2_ for SAHS detection. The NF signal was recorded by a thermistor while SpO_2_ was recorded by a finger pulse oximeter and the sampling rate of both was 8 Hz.

### Study design

**Fig. 1:**
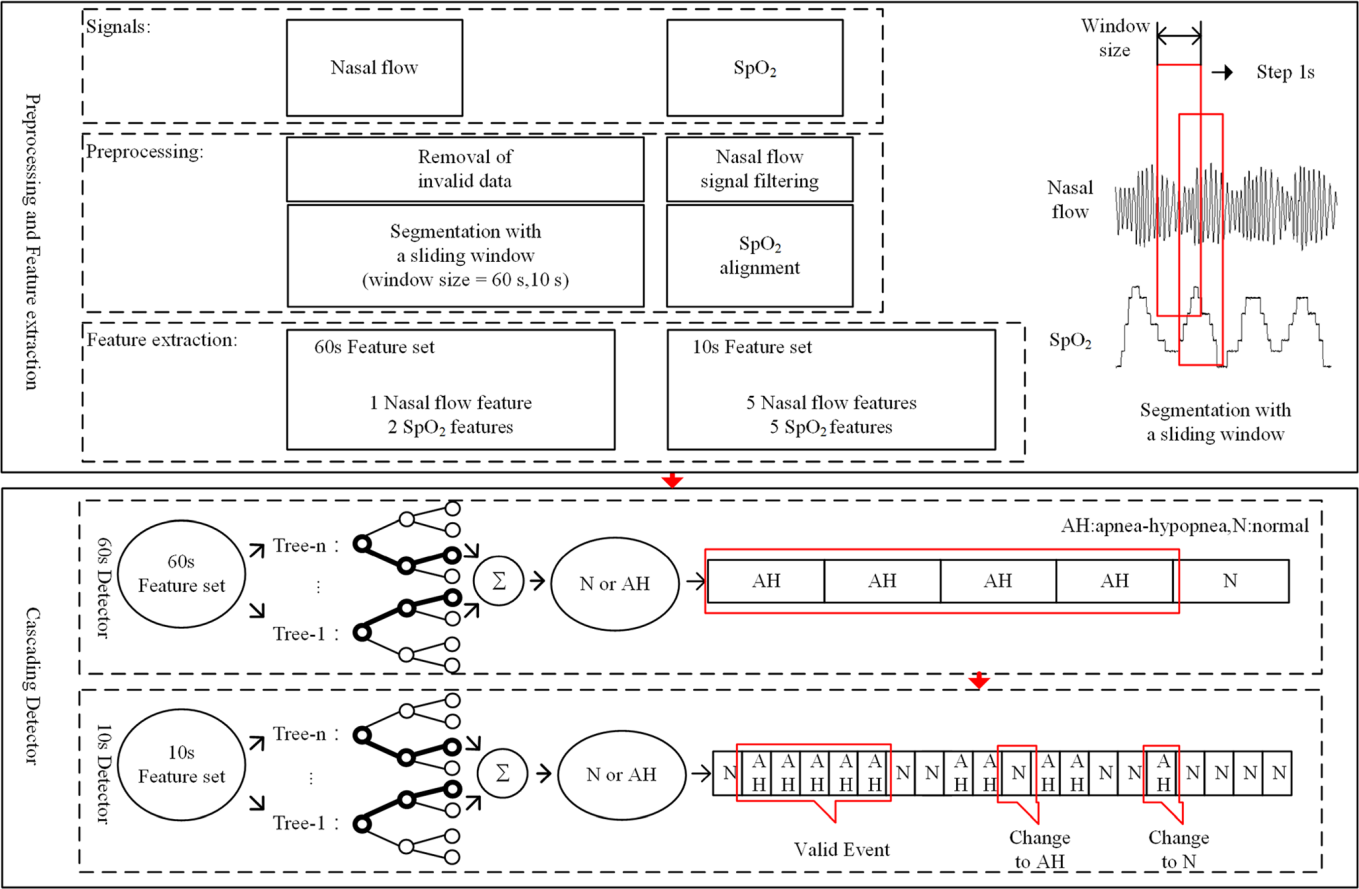
Design of cascading detection model based on AH event detection

The cascading detection model based on AH event detection is shown in Fig. 1. It comprises the following main steps: (1) removal of invalid data, NF signal filtering, segmentation with a sliding window, and SpO_2_ alignment; (2) extraction of a specific feature set from each segment; 3) the cascading detection model predicts each segment and outputs a sequence of segments’ results; (4) the event detector corrects the invalid results in the sequence and calculates the AHI.

### Signal preprocessing

Signal preprocessing comprises the following four steps: (1) removal of invalid data. Any SpO_2_ values lower than 50% were considered to be artifacts and removed from the analysis (5.6% of the data). (2) NF signal filtering—a four-point sliding average filter and a third-order Butterworth high-pass filter with a cutoff frequency of 0.05 Hz were used to prevent high-frequency noise caused by artifacts and baseline drift in NF signal. (3) Segmentation—the original signals were segmented using a 60-s window and a 10-s window, respectively. In both cases, the step was set to 1 s. All segments were categorized into two classes: AH and N according to the annotations. The segments containing more than 5 s of AH events were labeled as class AH. Other cases were labeled as class N. (4) SpO_2_ alignment. As SpO_2_ responds slowly to AH events [18], a time advance of *τ* s (0 < *τ* < 30) was applied in SpO_2_. The results showed that the model performed best with *τ* set to 23 s. After preprocessing, the number of extracted segments were 487,974 (AH, *N* = 44,476: 443,498).

### Feature extraction

#### NF feature set

According to the AASM definition of AH events, the amplitude of NF provides important information. Therefore, we first extracted the maximum and minimum points from each NF segment. Then, the tidal volume per breath *Ft* was calculated as the difference between two adjacent extreme points. The mean, standard deviation, and range of the tidal volume (*F*mean, *F*std, *F*ran) were extracted within each segment. Besides, we calculated the maximum value of the tidal volume every 30 s using Eq. (1):

1 $$ {Fb}_i=\max \left\{{Ft}_{i-30},{Ft}_{i-29},\dots, {Ft}_{i-1}\right\} $$

where *Ft*_*i*_ represents the tidal volume values in the *i*th segment. *Fb*_*i*_ represents the maximum tidal volume value in 30 s before the *i*th segment. The number of breaths with tidal volume drops by more than 30%, 70% from *Fb* were calculated within each segment and denoted as *Fha*, *Fap*. And the number of breaths with tidal volume above 85% of *Fb* was also calculated and denoted as *Fnor*. In addition, the ratios of them to the total number of breaths (*Fhap*, *Fapp*, *Fnorp*) within each segment were calculated. Besides, owing to the cessation of breathing, there will be fluctuations in the breathing rate during AH events. One normal breath lasts for 3–5 s; energy will be concentrated with a peak in the corresponding frequency. As a result, we took the fourth statistical moment (*Fkur*) in 0.2–0.4 Hz of NF’s frequency spectrum as another feature.

#### SpO_2_ feature set

We first calculated the standard deviation and range coefficients of SpO_2_ (*Spstd*, *Spran*) in each segment. The tendency of SpO_2_ in each segment (*Spten*) was also calculated by using the last SpO_2_ value minus the first SpO_2_ value. The commonly used feature: time SpO_2_ stays below 90% [19, 20] was referred while we calculated the indices *Sp*92, *Sp*91 with thresholds set to 92% and 91%. Besides, the maximum SpO_2_ value (*Spbm*) and average SpO_2_ value (*Spba*) in every 30 s were computed. Then within each segment, the time SpO_2_ stays below 98% of *Spbm* and that below 98% of *Spba* were calculated and denoted as *Spdum*, *Spdua* respectively. Finally, the level of oxygen desaturation *Spldm* and *Splda* in each segment was calculated as Eqs. (2) and (3) show.

2 $$ { Sp ldm}_i={ Sp bm}_i-\mathrm{mean}\left\{{Sp}_i\right\} $$

3 $$ { Sp lda}_i={ Sp ba}_i-\mathrm{mean}\left\{{Sp}_i\right\} $$

where *i* represents the *i*th segment. *Sp*_*i*_ represents the SpO_2_ values in the *i*th segment. *Spbm*_*i*_, *Spba*_*i*_ represent the maximum and average SpO_2_ value in 30 s before the *i*th segment respectively. The total feature set is shown in Table 2.

**Table 2:** Features and their definitions

Index	Name	Definition
1	*Fmean*, *Fstd*, *Fran*	Average, standard deviation, and range of tidal volume
2	*Fha*, *Fhap*	Number of breaths with a reduction more than 30% in tidal volume and its ratio to total number of breaths
3	*Fap*, *Fapp*	Number of breaths with a reduction more than 70% in tidal volume and its ratio to total number of breaths
4	*Fnor*, *Fnorp*	Number of breaths with a reduction less than 15% in tidal volume and its ratio to total number of breaths
5	*Fkur*	Fourth statistical moment in 0.2–0.4 Hz of NF’s frequency spectrum
6	*Spstd*, *Spran*	Standard deviation and range of SpO_2_
7	*Spten*	Tendency of SpO_2_
8	*Spdum*, *Spdua*	Duration of SpO_2_ desaturation
9	*Spldm*, *Splda*	Level of SpO_2_ desaturation
10	*Sp*92, *Sp*91	Duration of SpO_2_ staying below 92 and 91%

### Design of cascading detector

The cascading detector contained two parts. The first was a random forest consisting of 10 CARTs for the prediction of 60 s segments. This could screen out most of the N segments while retaining the AH segments. The second part was a random forest consisting of 20 CARTs for the prediction of 10 s segments. Based on the results of the 60-s detector, the 10-s detector was able to detect AH events more precisely.

Note that the 60 s detector was trained using a feature set composed of features 2, 6, and 8 in Table 2 in order to improve the training speed. The results indicated that there was almost no effect on the performance. Owing to the imbalance in the number of AH and N segments, the weights for the two classes in CARTs were set to inverse ratio of their numbers.

A twofold cross-validation was used in the test. Each time, half of the segments were used for training with the remaining half used for testing. The cascading detector output the sequence composed of the prediction results of the 10 s segments. The detector was trained on a computer with an i5-7600k CPU and 8 G RAM.

### Design of event detector

The sequence predicted by the cascading detector was then fed into the event detector to correct invalid results following two rules; (1) Only more than 10 consecutive segments classified as AH were considered to be one valid AH event. As the original data were segmented by a 10-s window, and one AH event lasts at least 10 s, so one AH event corresponded to at least 10 consecutive AH segments. Any segment which did not meet the rule was modified to class N. (2) The number of segments classified as N between two valid AH segments was supposed to be more than five. This was also determined by the way of data segmentation. Any segment that did not meet the rule was reset to class AH.

## Results

The cascading detection model was able to estimate AHI and provide the time information for each AH event. We analyzed its performance with respect to two aspects: segments and AHI.

### Segment analysis

The prediction results for the segment-by-segment analysis are shown in Table 3. The cascading detection model achieved an accuracy of 88.3%, a sensitivity of 75.2%, and a specificity of 89.6% for 487,974 test segments.

**Table 3:** Results for segments

		Reference		ACC (%)	SEN (%)	SPE (%)
		AH	*N*			
Estimated	AH	33,429	46,247	88.3	75.2	89.6
	N	11,047	397,251			

*ACC* accuracy, *SEN* sensitivity, *SPE* specificity

**Fig. 2:**
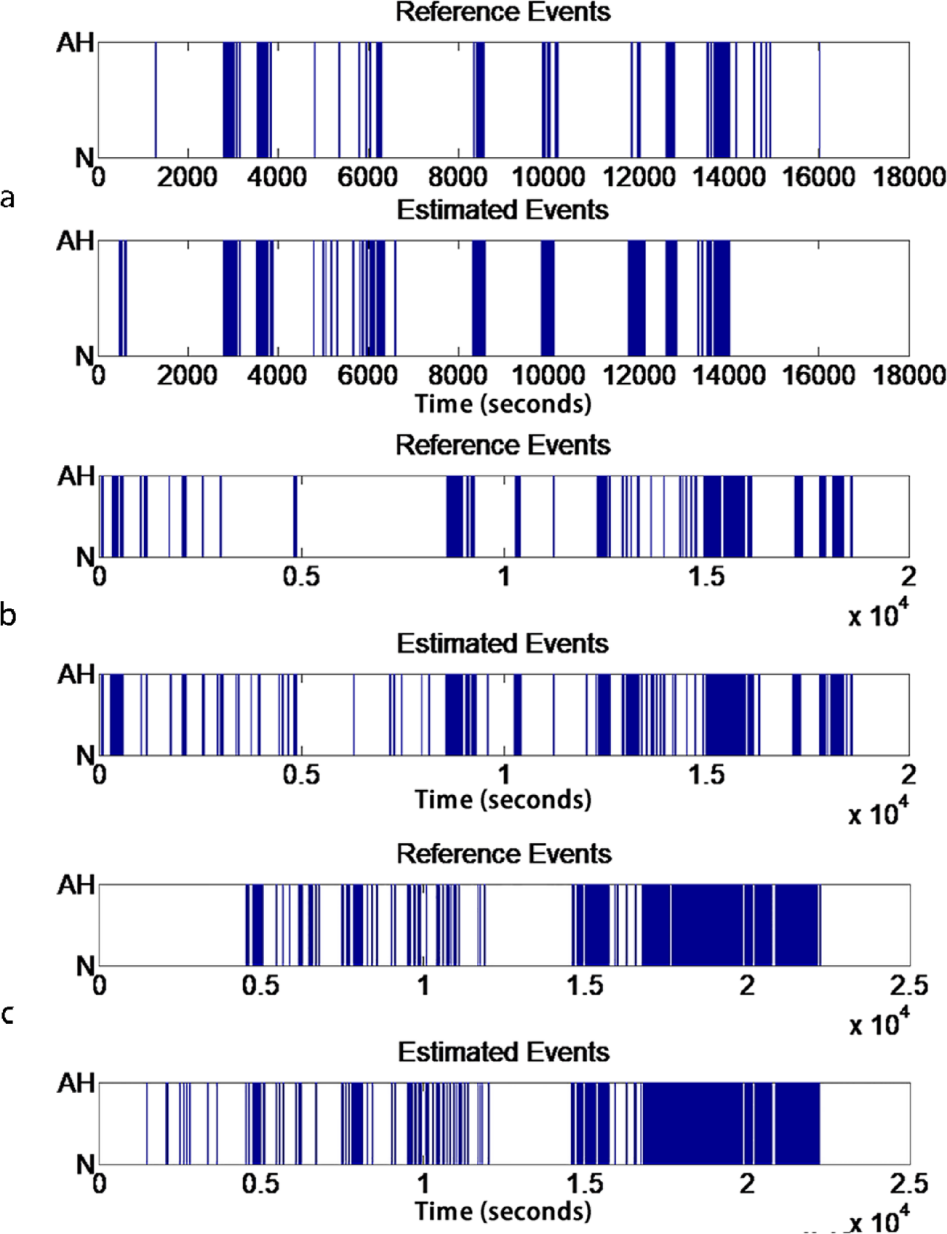
**a** AH event estimation result for mild-SAHS. **b** AH event estimation result for medium-SAHS. **c** AH event estimation result for severe-SAHS

Figure 2 displays the AH event estimation results for one mild-SAHS, medium-SAHS, and severe-SAHS subject. For the mild-SAHS subject, the accuracy, sensitivity and specificity were 92.9%, 80.6%, and 94.0%. The corresponding values for the medium-SAHS subject were 92.2%, 82.2%, and 93.6% while 86.7%, 81.1%, and 88.2% for the severe one.

### AHI analysis

**Fig. 3:**
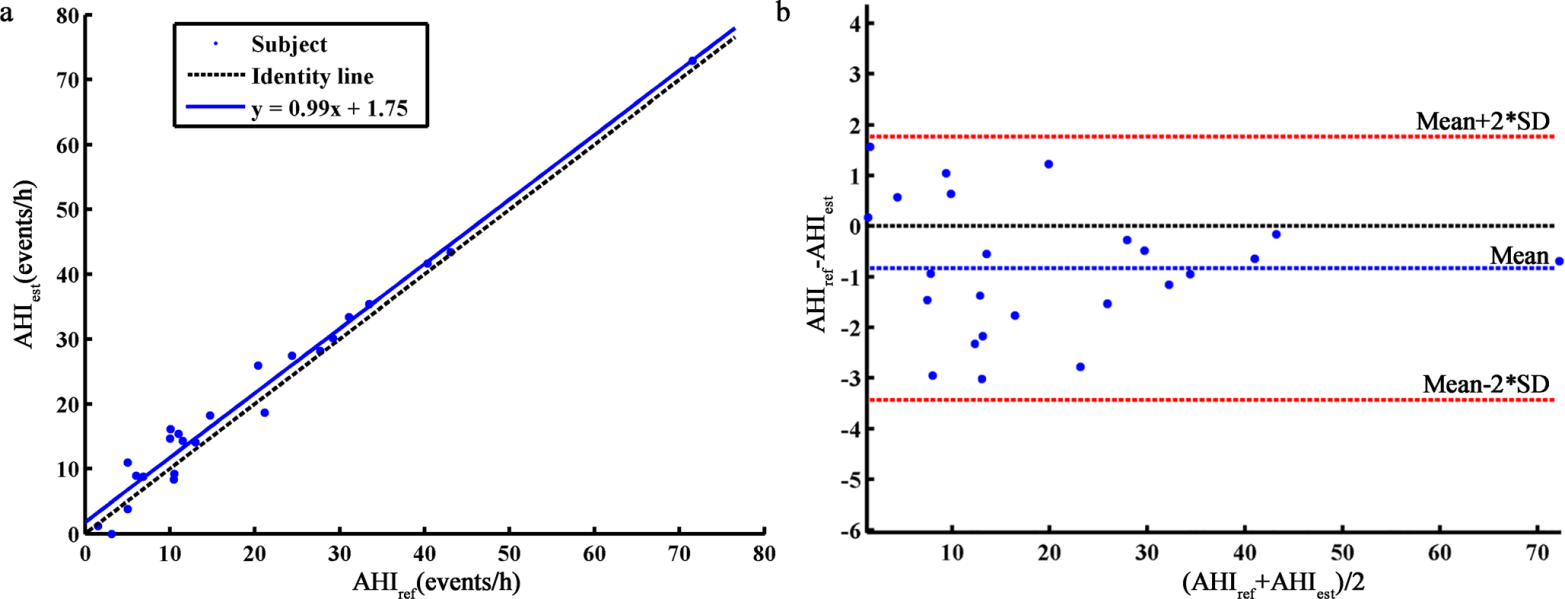
**a** Scatter plot of AHI_est_ and AHI_ref_. **b** Bland–Altman plot of AHI_est_ and AHI_ref_

Figure 3(a) shows a scatter plot of the AHI (AHI_est_) estimated by the model and the AHI (AHI_ref_) determined from PSG. The solid line fitted shows a high correlation (Pearson’s correlation coefficient 0.99, *p* < 0.01) between AHI_est_ and AHI_ref_. Figure 3(b) shows the Bland–Altman plot of AHI_est_ and AHI_ref_. The average error of AHI_est_ and AHI_ref_ was − 0.8 events/h, and the error range was − 3.4 to 1.8 events/h (95% confidence interval).

Table 4 compares the number of AH events, duration of AH events and AHI values for the cascading model and scored by PSG. Table 5 summarizes the classification results for SAHS severity. The mean values for sensitivity, specificity, PPV, and accuracy were 100.0%, 91.1%, 86.7%, and 94.4%, respectively, for AHI thresholds of 5, 15, and 30 events/h. Besides, the kappa coefficient for diagnosis of SAHS severity was 0.76.

**Table 4:** Comparison of number of AH events, duration of AH events, and AHI predicted by cascading model with PSG (mean ± standard deviation)

Group	Number of AH events		Duration of AH events (min)		AHI (events/h)	
	Reference	Estimated	Reference	Estimated	Reference	Estimated
Non	17.0 ± 7.0	9.0 ± 8.3	4.6 ± 2.3	6.2 ± 4.6	3.2 ± 1.4	1.7 ± 1.6
Mild	61.5 ± 19.4	83.0 ± 37.2	18.8 ± 6.2	29.6 ± 7.9	9.9 ± 2.8	12.1 ± 4.2
Moderate	132.2 ± 18.7	155.0 ± 23.6	42.5 ± 14.2	54.3 ± 15.5	24.6 ± 3.5	27.5 ± 5.4
Severe	193.2 ± 63.2	200.2 ± 63.8	61.3 ± 21.2	72.2 ± 30.8	43.9 ± 14.5	45.3 ± 14.3

**Table 5:** SAHS severity classification and diagnostic performance

		Determined from PSG					AHI cutoff (\)			
		Non	Mild	Moderate	Severe		≥ 5	≥ 15	≥ 30	AVE
Estimated	Non	2	0	0	0	SEN (%)	100.0	100.0	100.0	100.0
	Mild	0	9	0	0	SPE (%)	100.0	78.6	94.7	91.1
	Moderate	0	3	4	0	PPV (%)	100.0	76.9	83.3	86.7
	Severe	0	0	1	5	ACC (%)	100.0	87.5	95.8	94.4

*ACC* accuracy, *SEN* sensitivity, *SPE* specificity, *PPV* positive predictive value

## Discussion

We proposed a cascading detection model that could predict AHI based on AH event detection. Compared with PSG, only NF and SpO_2_ were used. Previously, the original signals were commonly cut into 60 s segments for AH event detection [2, 7, 10, 11]. However, the detection of AH events may not be precise based on 60 s segment analysis because it can only determine whether there was AH in the segment, while, may make mistakes for the segments containing multiple AH events and lead to an error in AHI estimation. Therefore, some researchers [3, 9] cut the signals into shorter segments for detection. However, it is difficult to extract effective features from a segment shorter than 10 s, because there will be no more than five complete breaths in one segment in most cases. As a result, we proposed a cascading detection model composed of a 60-s detector and a 10-s detector to predict AH events precisely. Table 3 shows the classification results for the segments. Notably, the model tended to make false positive errors. In approximately 12.1% of these errors, the amplitude of NF signal decreased by more than 30% from previous event baseline accompanied with a SpO_2_ desaturation, however no arousal or nasal pressure signals were adopted for identifying hypopneas. Therefore, these segments may be mistaken for class AH.

As illustrated in Fig. 3, AHI_est_ showed high correlation with AHI_ref_ (Pearson correlation coefficient 0.99, *p* < 0.01). The performance of the model also showed good consistency among different subjects. On the other hand, AHI_est_ was slightly higher than AHI_ref_. Consequently, SAHS severity was overestimated for four subjects; for the remaining 20 subjects, the model gave the correct prediction (Table 5). The kappa coefficient of the cascading detection model for diagnosis of SAHS severity was 0.76, indicating that this method represents a powerful screening tool for SAHS.

We also tested the speed of the cascading detection model. Training required 24.7 s, while only 20.3 s was needed to provide results for all segments and to predict AHI for all 24 subjects. It took 41.6 μs to predict one segment and 0.85 s to diagnose one subject on average. This implies that the model could be used for real-time AH event detection.

As Table 6 shows, our method exhibited a good sensitivity but not very good specificity compared with other studies. That is mainly because excursions in NF is not as prominent as those in nasal pressure signal during hypopnea [16] thus decrease the event detection performance. Nasal pressure signal or a combination of NF and nasal pressure signal will be taken into study in future to improve this. More importantly, the model could not only predict the severity of SAHS but could also provide time information for each AH event. Furthermore, compared with other methods such as convolutional neural networks, a smaller number of hyperparameters and less computation were required by our random forest based approach, and the CARTs provided better interpretability for clinical detection.

**Table 6:** Comparison with other studies

Related work	Method	Signal	AHI cutoff	ACC (%)	SEN (%)	SPE (%)
Choi et al. [3]	Convolutional neural networks	Nasal pressure	5	96.2	100.0	84.6
			15	92.3	98.1	86.5
			30	96.2	96.2	96.2
Gonzalo et al. [4]	AdaBoost-Linear discriminant analysis	Nasal flow	5	86.5	87.1	80.0
			15	81.0	85.9	72.9
			30	82.5	74.2	90.6
Da Woon Jung et al. [7]	Rule-based	SpO_2_	5	97.8	98.6	94.4
			10	96.7	98.4	92.9
			15	95.7	96.4	94.6
			30	96.7	97.1	96.5
Our study	Cascade of random forests	Nasal flow and SpO_2_	5	100.0	100.0	100.0
			15	87.5	100.0	78.6
			30	95.8	100.0	94.7

*ACC* accuracy, *SEN* sensitivity, *SPE* specificity

However, there were some limitations to this study. First, we did not further classify AH events into apnea events and hypopnea events. Second, the model was not tested in an online environment. We hope to confirm the usability of our method online in the future. Third, the model was not able to distinguish central and obstructive events because no ribcage or abdominal movement signals for identifying central events from obstructive events were adopted in this study. Finally, no electroencephalography was adopted in this algorithm, thus sleep and awake time were not evaluated in this study.

## Conclusion

The purpose of this study was to propose a model for real-time detection of AH events. Based on the morphological features of NF and SpO_2_, the cascade of a 60-s detector and 10-s detector could not only predict AH events, but could also provide time information for each AH event. Compared with previous research, the cascading detection model based on random forests provides better interpretation with reduced computational complexity. Therefore, it is expected to be an effective tool for SAHS diagnosis.
